# Multimodal mechano-microscopy reveals mechanical phenotypes of breast cancer spheroids in three dimensions

**DOI:** 10.1063/5.0213077

**Published:** 2024-09-09

**Authors:** Alireza Mowla, Matt S. Hepburn, Jiayue Li, Danielle Vahala, Sebastian E. Amos, Liisa M. Hirvonen, Rowan W. Sanderson, Philip Wijesinghe, Samuel Maher, Yu Suk Choi, Brendan F. Kennedy

**Affiliations:** 1 BRITElab, Harry Perkins Institute of Medical Research, QEII Medical Centre, Nedlands, WA 6009, Australia; 2 Department of Electrical, Electronic and Computer Engineering, School of Engineering, The University of Western Australia, Perth, WA 6009, Australia; 3 Institute of Physics, Faculty of Physics, Astronomy and Informatics, Nicolaus Copernicus University in Toruń, Grudziadzka 5, 87-100 Torun, Poland; 4 School of Human Sciences, The University of Western Australia, Perth, WA 6009, Australia; 5 Centre for Microscopy, Characterisation and Analysis, The University of Western Australia, Perth, WA 6009, Australia; 6 Centre of Biophotonics, SUPA, School of Physics and Astronomy, University of St Andrews, St Andrews KY16 9SS, United Kingdom

## Abstract

Cancer cell invasion relies on an equilibrium between cell deformability and the biophysical constraints imposed by the extracellular matrix (ECM). However, there is little consensus on the nature of the local biomechanical alterations in cancer cell dissemination in the context of three-dimensional (3D) tumor microenvironments (TMEs). While the shortcomings of two-dimensional (2D) models in replicating *in situ* cell behavior are well known, 3D TME models remain underutilized because contemporary mechanical quantification tools are limited to surface measurements. Here, we overcome this major challenge by quantifying local mechanics of cancer cell spheroids in 3D TMEs. We achieve this using multimodal mechano-microscopy, integrating optical coherence microscopy-based elasticity imaging with confocal fluorescence microscopy. We observe that non-metastatic cancer spheroids show no invasion while showing increased peripheral cell elasticity in both stiff and soft environments. Metastatic cancer spheroids, however, show ECM-mediated softening in a stiff microenvironment and, in a soft environment, initiate cell invasion with peripheral softening associated with early metastatic dissemination. This exemplar of live-cell 3D mechanotyping supports that invasion increases cell deformability in a 3D context, illustrating the power of multimodal mechano-microscopy for quantitative mechanobiology *in situ*.

## I. INTRODUCTION

During metastasis, the main cause of cancer-related deaths, cancer cells must overcome mechanical challenges to invade distant tissues and organs.^1^ Understanding the physiochemical interactions between the tumor microenvironment (TME) and cancer cells is imperative in developing novel anticancer treatments.^2^ Although it is known that various cancer types influence metastasis through a range of mechanotransduction pathways,^3^ the lack of tools to measure sub-cellular elasticity (i.e., Young's modulus) in three-dimensional (3D) *in vitro* tumor models, such as widely used cell spheroid systems, impedes current attempts to link functional biochemistry to the local biomechanical phenotypes of cancer.^4^ Atomic force microscopy (AFM), a gold standard in mechanobiology, does not offer depth penetration.^5–7^ Micropipette aspiration, parallel-plate compression,^5^ and deformability cytometry^8^ provide bulk mechanical measurements of entire cells. Optical tweezers^5^ and calibrated biosensors, such as those based on fluorescence resonance energy transfer,^9,10^ magnetically responsive microdroplets,^11^ or thermally responsive microgels,^12^ provide invasive point measurements.^5^ 3D traction force microscopy has shown great promise, but it provides measurements of forces exerted by cells, rather than a mechanical property, such as Young's modulus, of cells and their surrounding environment.^13^ Optical elastography techniques, particularly optical coherence elastography (OCE) and Brillouin microscopy, have shown promise in biomechanical characterization of cells, biomaterials, and tissues, as well as advancement toward clinical applications.^14–16^ However, optical elastography techniques have key limitations. For example, OCE, which is based on optical coherence tomography (OCT), is unable to achieve sub-cellular elasticity resolution in both individual cells^17–19^ and cell spheroid models.^20^ Brillouin microscopy can achieve sub-cellular resolution, however, it measures longitudinal modulus in the gigahertz regime, which is challenging to relate to Young's modulus, the most commonly used mechanical modulus, independent of water content and, also, has a limited penetration depth (∼100–200 *μ*m) in opaque samples.^21^ Furthermore, as it is becoming increasingly clear that the interplay between structure, biomechanics, and biochemistry is central to the onset and progression of cancer,^22^ it is likely that a multimodal imaging platform is required to enable future breakthroughs in mechanobiology, yet no such platform currently exists.

The paucity of tools available to non-invasively map 3D Young's modulus of TMEs leaves the mechanical phenotype of metastasis and the role of extracellular matrix (ECM) elasticity subject to debate.^5^ Although metastatic cells are usually reported to be softer than their nonmalignant counterparts,^23^ studies also suggest that they can instead stiffen during invasion.^24^ Furthermore, while there is a positive correlation between increased ECM elasticity and cell invasion,^25^ it has also been reported that a stiff ECM indirectly inhibits cancer cell metastasis by modulation of mesenchymal stem cell secretions following mechanically induced differentiation.^26^ These studies typically rely on point or 2D mechanical characterization of isolated cell systems that are well known to exhibit behaviors atypical of *in vivo* settings. Here, we overcome this major limitation by developing multimodal mechano-microscopy, an imaging platform that is capable of non-invasively quantifying Young's modulus with sub-cellular resolution, co-registered with sub-cellular function provided by integrated confocal fluorescence microscopy (CFM), as well as 3D micro-scale structure from optical coherence microscopy (OCM). Mechano-microscopy is a low-coherence interferometric technique that provides a penetration depth of ∼500 *μ*m in scattering samples.^27^ Mechano-microscopy uses phase-sensitive OCM to map nano- to micro-scale axial deformation induced in the sample using a piezoelectric actuator with spatial resolution of 0.5 × 0.5 × 1.4 *μ*m^3^ (*xyz*) in air. In post-processing, a mechanical model based on continuum mechanics is used to convert experimentally measured deformation into a high-resolution map of 3D Young's modulus. Here, we demonstrate the capability of multimodal mechano-microscopy by performing a study on non-metastatic and metastatic cancer cell spheroids. Through close correspondence between structural OCM, Young's modulus, and CFM images, our results confirm that the mechanical phenotype of metastasizing cancer cells encapsulated in stiff and soft 3D TME-mimicking hydrogels, representative of primary and invasive sites,^28^ respectively, is mediated by the ECM stiffness. We believe that the new capability of multimodal mechano-microscopy, demonstrated here, is primed for the next generation of discoveries in cancer mechanobiology.

## II. RESULTS

**FIG. 1.**
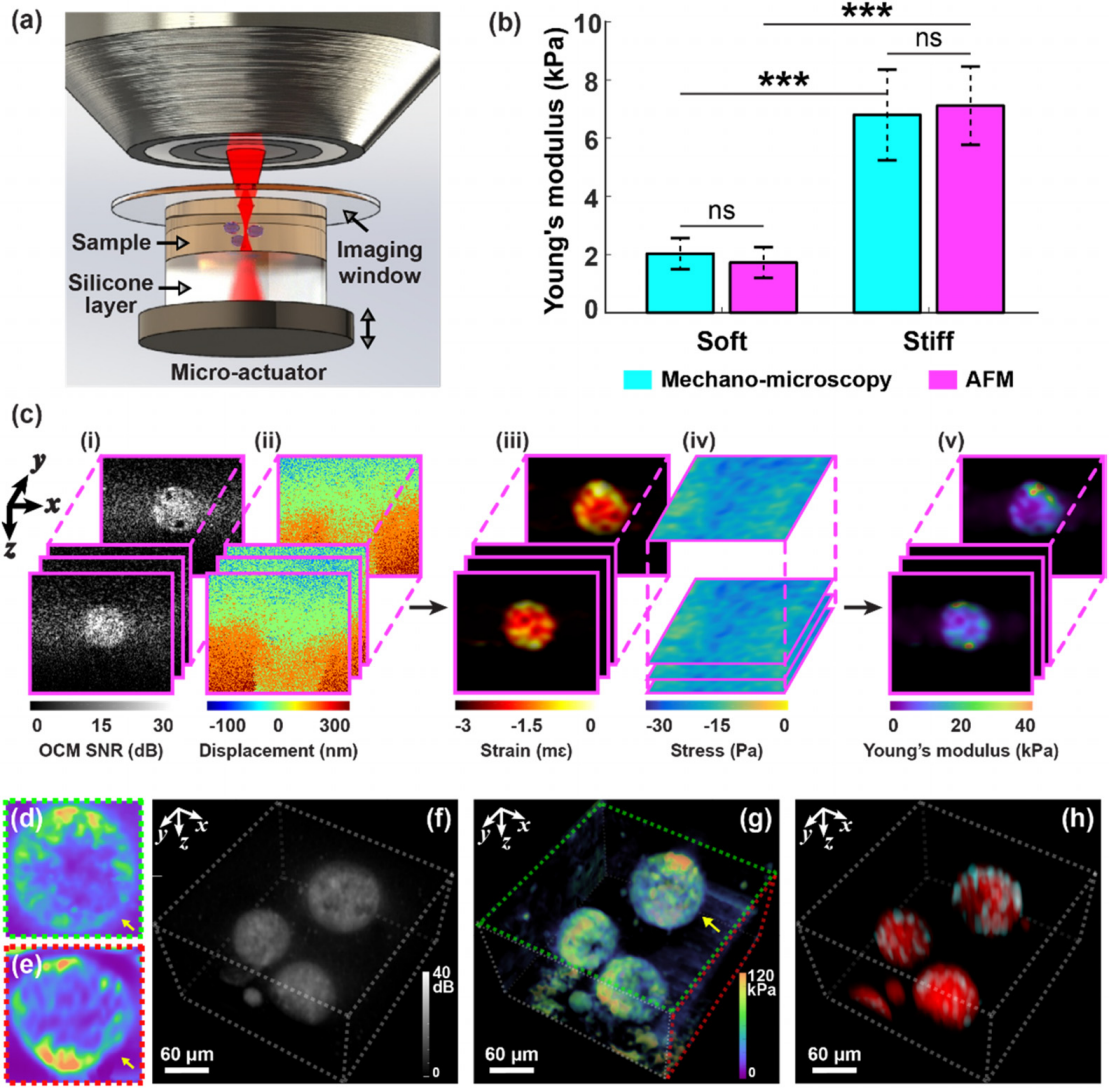
Multimodal mechano-microscopy. (a) Schematic of the sample setup. (b) Young's modulus of soft and stiff blank GelMA samples (*n* = 6 in each case) measured using mechano-microscopy (volumetric measurements) and AFM (surface measurements). Error bars represent standard deviation across independent sample measurements. A two-sample *t*-test was used to assess the statistical significance of each population, showing no statistical significance (ns) between the measurements of mechano-microscopy and AFM in both soft (*p* = 0.30 > 0.05) and stiff (*p* = 0.71 > 0.05) GelMA characterization, and statistical significance in the measured Young's modulus between the soft and stiff GelMA samples using mechano-microscopy (*p* = 1.03 × 10^−6^ < 0.001, ^***^) and AFM (*p* = 3.68 × 10^−6^ < 0.001, ^***^), respectively. (c) Image processing of mechano-microscopy illustrated using multiple B-scans of a non-metastatic cell spheroid with a 158 × 133 mm^2^ field-of-view (*xz*) at different *y* locations, including (i) OCM SNR, (ii) displacement, (iii) strain, (iv) stress, and (v) Young's modulus. (d) *En face* and (e) B-scan Young's modulus of a non-metastatic cell spheroid over a 120 × 120 mm^2^ field-of-view. (f)–(h) Volumetric maps of non-metastatic cell spheroids acquired by multimodal mechano-microscopy, where (f) OCM SNR, (g) Young's modulus, and (h) fluorescence labeled with nuclear (cyan) and membrane (red) fluorescent dyes. Yellow arrows indicate the same spheroid in (d), (e), and (g).

Mechano-microscopy quantifies the local micro-scale mechanical properties of a sample using the principles of compression OCE.^29,30^ As illustrated in the schematic in Fig. 1(a), the sample and a compliant silicone layer^31^ are placed between a microscope coverslip used as an imaging window and a piezoelectric actuator that imparts sequential micro-scale compression to the system. Figure 1(c) illustrates the image processing in mechano-microscopy (details are presented in Sec. IV A) for the case of a non-metastatic spheroid. Briefly, under micro-scale compression, the structure [Fig. 1(c-i)] and axial displacement [Fig. 1(c-ii)] of the sample are imaged using OCM with spatial resolution of 0.5 × 0.5 × 1.4 *μ*m^3^ (*xyz*) in air. Local strain [Fig. 1(c-iii)] in the sample is calculated as the gradient of axial displacement with depth, and local stress is converted from local strain in the layer through the pre-characterized stress–strain relationship of the layer and is then spatially mapped onto the layer–sample interface to generate a 2D stress map [Fig. 1(c-iv)]. By assuming uniaxial stress, the local Young's modulus [Fig. 1(c-v)] is estimated as the ratio of layer stress to sample strain at a system resolution of 5 × 5 × 15 *μ*m^3^ (*xyz*). Multimodal mechano-microscopy further records co-localized sample structure from the OCM signal-to-noise ratio (SNR) and fluorescence from CFM. We first validate our measurements against AFM on blank, homogeneous gelatin methacryloyl (GelMA) samples (*n* = 6) that were photopolymerized for 15 and 60 s, respectively, hereby referred to as soft and stiff hydrogels. Figure 1(b) shows that both mechano-microscopy and AFM can differentiate soft and stiff hydrogels with statistical significance (*p* < 0.001), suggesting that Young's modulus of the stiff hydrogels is ∼3.5× greater than that of the soft hydrogels, through close correspondence between the two techniques, indicated by no statistical significance (ns) between both measurements. The slight discrepancy between the measurements of mechano-microscopy and AFM is likely due to the different mechanical loading used (i.e*.,* compression vs indentation). Such inter-tool variability is expected and well documented for biological samples on this scale.^32^ Figures 1(d) and 1(e) exemplify *xy* (*en face*) and *yz* cross-sectional images of the local Young's modulus of a living breast cancer (MCF-7) spheroid encapsulated in stiff GelMA. Figures 1(f)–1(h) present the full complement of imaging capabilities with 3D visualizations of structure, Young's modulus, and fluorescence over a volume of 300 × 300 × 160 *μ*m^3^ (*xyz*). The cyan and red regions in Fig. 1(h) represent nuclei and cell membranes, labeled with live-cell compatible dyes Hoechst 33342 and CellMask Green, respectively (details are presented in Sec. IV F).

**FIG. 2.**
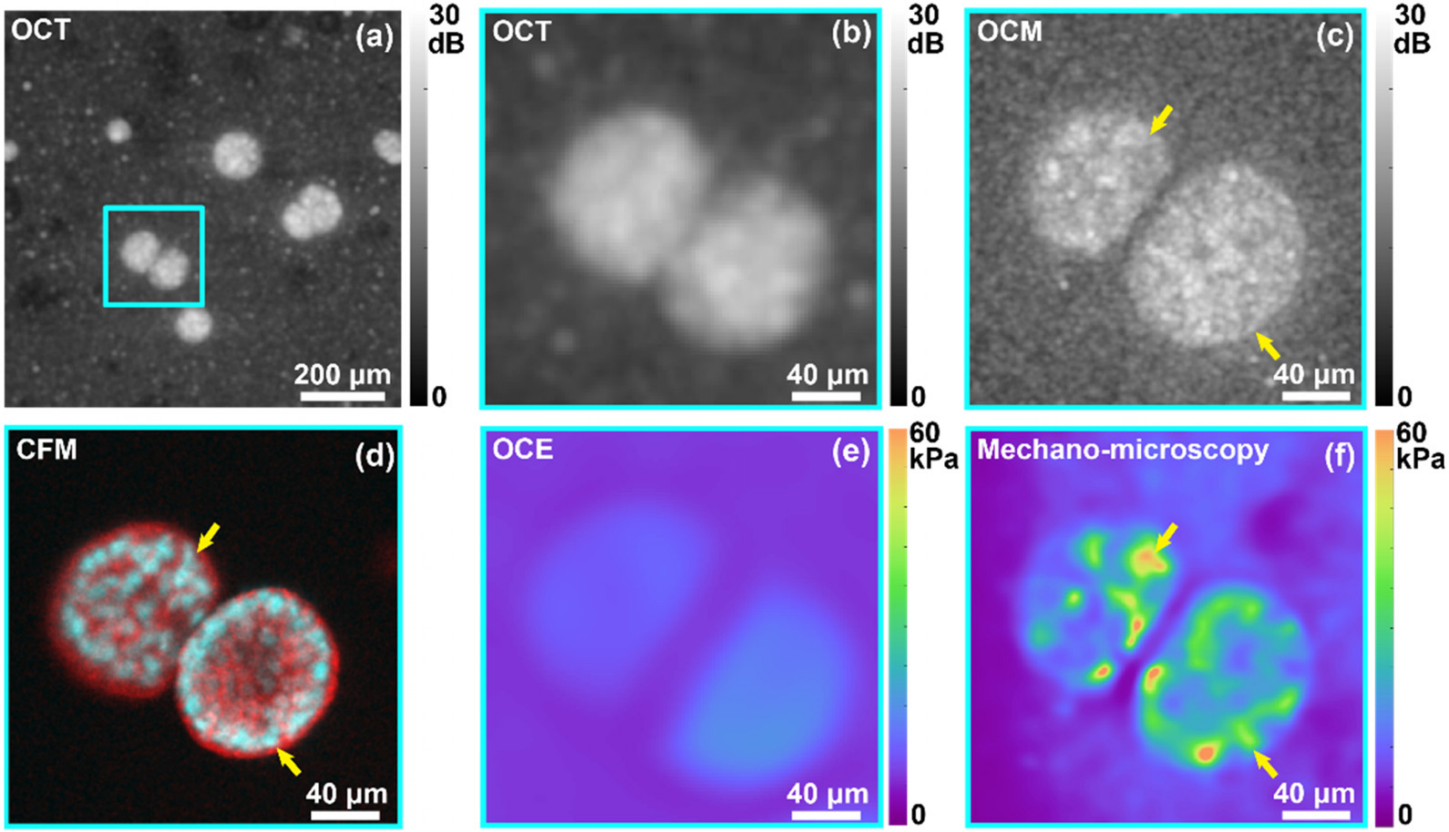
OCE vs mechano-microscopy on non-metastatic breast cancer cell spheroids in GelMA. (a) *En face* (*xy*) OCT image of the spheroids. The cyan box indicates the location of spheroids of interest. The corresponding magnified (b) OCT SNR and (e) Young's modulus using OCE in comparison to the co-registered (c) OCM SNR, (f) Young's modulus, and (d) fluorescence labeled with nuclear (cyan) and membrane (red) fluorescent dyes using mechano-microscopy. Yellow arrows in (c), (d), and (f) highlight nuclei in the cell spheroids.

In Fig. 2, a comparison is presented between OCE (details are presented in Sec. IV C)^17^ and multimodal mechano-microscopy performed on breast cancer (MCF-7) cell spheroids encapsulated in GelMA, similar to the spheroid presented in Figs. 1(d)–1(h). In Fig. 2(b), a magnified OCT image is presented that corresponds to the region highlighted by the cyan box in Fig. 2(a). From the co-registered fluorescence image presented in Fig. 2(d), it is evident that the higher resolution provided by OCM [Fig. 2(c)] reveals structures not visible in the OCT image. In particular, the yellow arrows in Figs. 2(c) and 2(d) highlight sub-cellular structures, likely corresponding to the cell nuclei, which present as regions of lower OCM SNR, due to relatively low and less heterogenous refractive index and mass density than cytoplasm.^33–35^ It is challenging to identify these structures in the OCT image in Fig. 2(b). The Young's modulus images, corresponding to OCE and mechano-microscopy, respectively, are presented in Figs. 2(e) and 2(f), respectively. It is evident that, for these spheroids, it is not possible to reveal intra-spheroidal structures using OCE, with a relatively uniform Young's modulus presented throughout the spheroids. Mechano-microscopy [Fig. 2(f)], on the other hand, reveals rich contrast within the spheroids. In particular, a relatively high Young's modulus is evident around the perimeter of the spheroid at the bottom right, which, through comparison with the CFM image [Fig. 2(d)] likely corresponds to regions with a denser concentration of cell nuclei, which is known as the stiffest organelle within a cell.^36^ We note that, due to the two spheroids located at different depths, the *en face* planes presented in Fig. 2 cross the bottom right spheroid at the middle, while the other at the top left was crossed at a plane closer to the top of the spheroid.

**FIG. 3.**
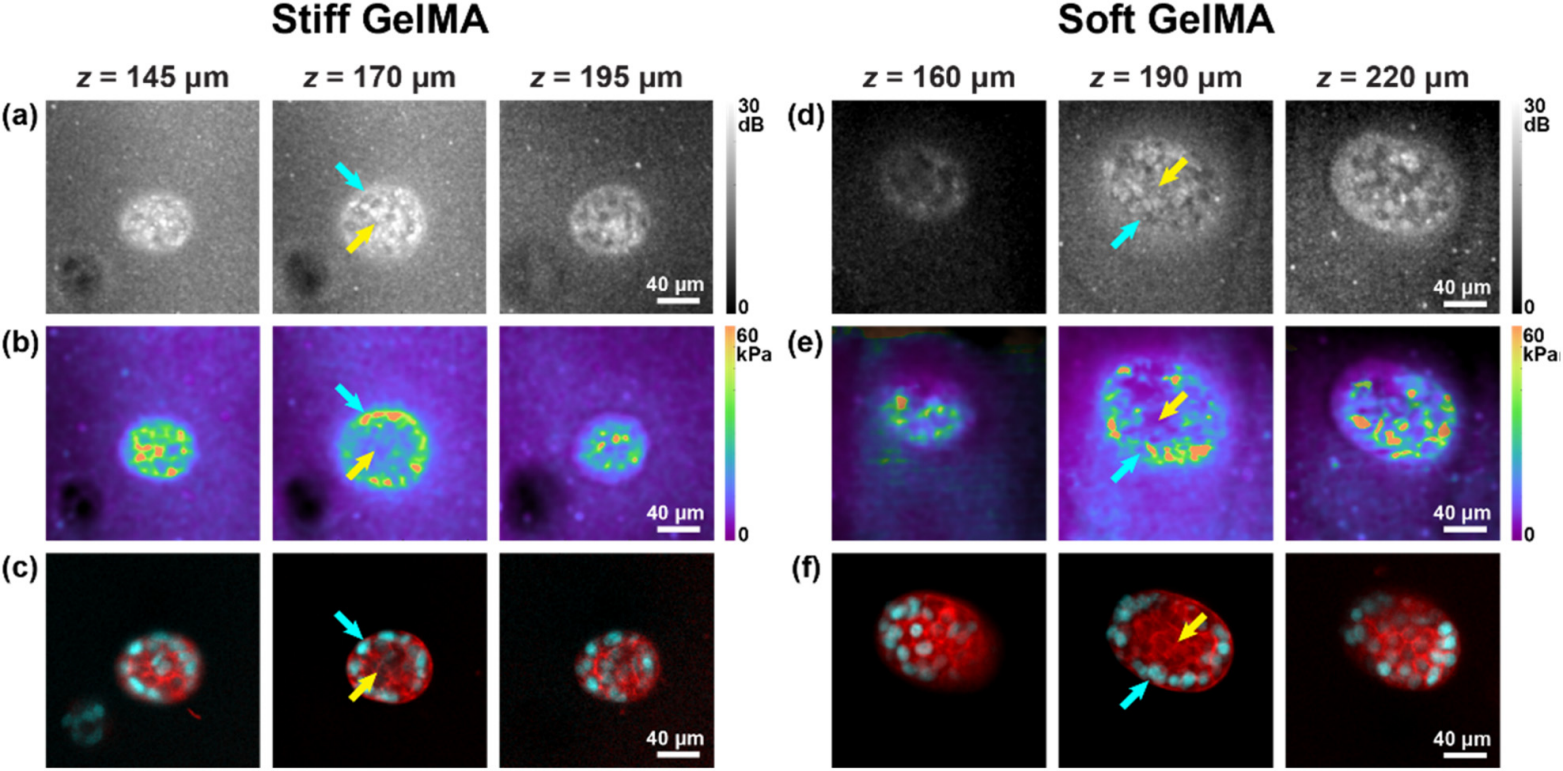
*En face* images of [(a) and (d) OCM SNR, [(b) and (e)] Young's modulus, and the co-registered [(c) and (f)] fluorescence labeled with nuclear (cyan) and membrane (red) fluorescent dyes, presented at three annotated depths, corresponding to the top, middle, and bottom cross-sectional planes of the cell spheroids. Yellow and cyan arrows in (a)–(f) indicate the core and periphery of the cell spheroids, respectively.

We demonstrate mechano-microscopy by investigating the mechanical phenotypes of non-metastatic (MCF-7) and metastatic (MDA-MB-231) cancer cell spheroids cultured in stiff (∼7 kPa) and soft (∼2 kPa) 3D GelMA hydrogels. The choice of hydrogel stiffnesses for spheroid experiments was made based on the consideration of biological relevance with human breast cancer, where the stiff and soft hydrogels were used to mimic the stiffness of *ex vivo* human breast primary and invasive cancer sites characterized by AFM in a previous study.^28^ In Fig. 3, examples of non-metastatic spheroids cultured in both the stiff and soft hydrogels are presented, representative of repeated independent samples (*n* = 6 for stiff and *n* = 4 for soft). *En face* (*xy*) cross sections of OCM [Figs. 3(a) and 3(d)], Young's modulus [Figs. 3(b) and 3(e)], and fluorescence image [Figs. 3(c) and 3(f)] are presented at three depths, where the middle plane represents the spheroid's central cross section, and the top and bottom planes were selected to be at the halfway point between the middle plane and the outer perimeter of the top and bottom of the spheroid, respectively. The fluorescence image suggests greater cell concentration in the spheroid periphery (cyan arrows) than in the core (yellow arrows).^37^ This has been observed previously at lower resolution using OCE^20^ and may be associated with the formation of growth-arrested clusters, which are similar to acini found *in vivo* in mammary glands.^38^ In the OCM images, as in Figs. 3(a) and 3(d), nuclei present as structures with low SNR, suggesting that they exhibit lower optical backscattering at the wavelengths used than the higher SNR observed in the cell body. In the Young's modulus images [Figs. 3(b) and 3(e)], elevated stiffness is again observed at the spheroid periphery. This is consistent across independent samples measurements in both stiff (core: 9.85 ± 5.42 kPa; periphery: 24.5 ± 11.9 kPa) and soft (core: 4.53 ± 1.84 kPa; periphery: 9.97 ± 4.62 kPa) hydrogels, which are summarized in Table I. The quantitative analysis at the core and periphery was determined using a segmentation method, which is described in Sec. IV G. This is likely because of increased deposition and remodeling of ECM proteins by breast cancer cells, including MCF-7 and MDA-MB-231, which has been studied previously,^39,40^ leading to potential self-compression of the tumor and the observed peripheral stiffening.^1^ The spheroid encapsulated in the soft hydrogel [Figs. 3(d)–3(f)] is larger than the one in the stiff hydrogel [Figs. 3(a)–3(c)], consistent with our previous study using OCE, which showed that spheroids grown in soft hydrogels have relatively larger volumes than those grown in stiff hydrogels.^20^

**TABLE I.** Quantitative comparison of non-metastatic and metastatic spheroids in both stiff and soft GelMA.

Young's modulus (kPa)	Non-metastatic		Metastatic	
	Core	Periphery	Core	Periphery
Stiff	9.85 ± 5.42	24.5 ± 11.9	9.22 ± 1.59	14.1 ± 3.06
Soft	4.53 ± 1.84	9.97 ± 4.62	11.8 ± 2.73	7.67 ± 0.71

**FIG. 4.**
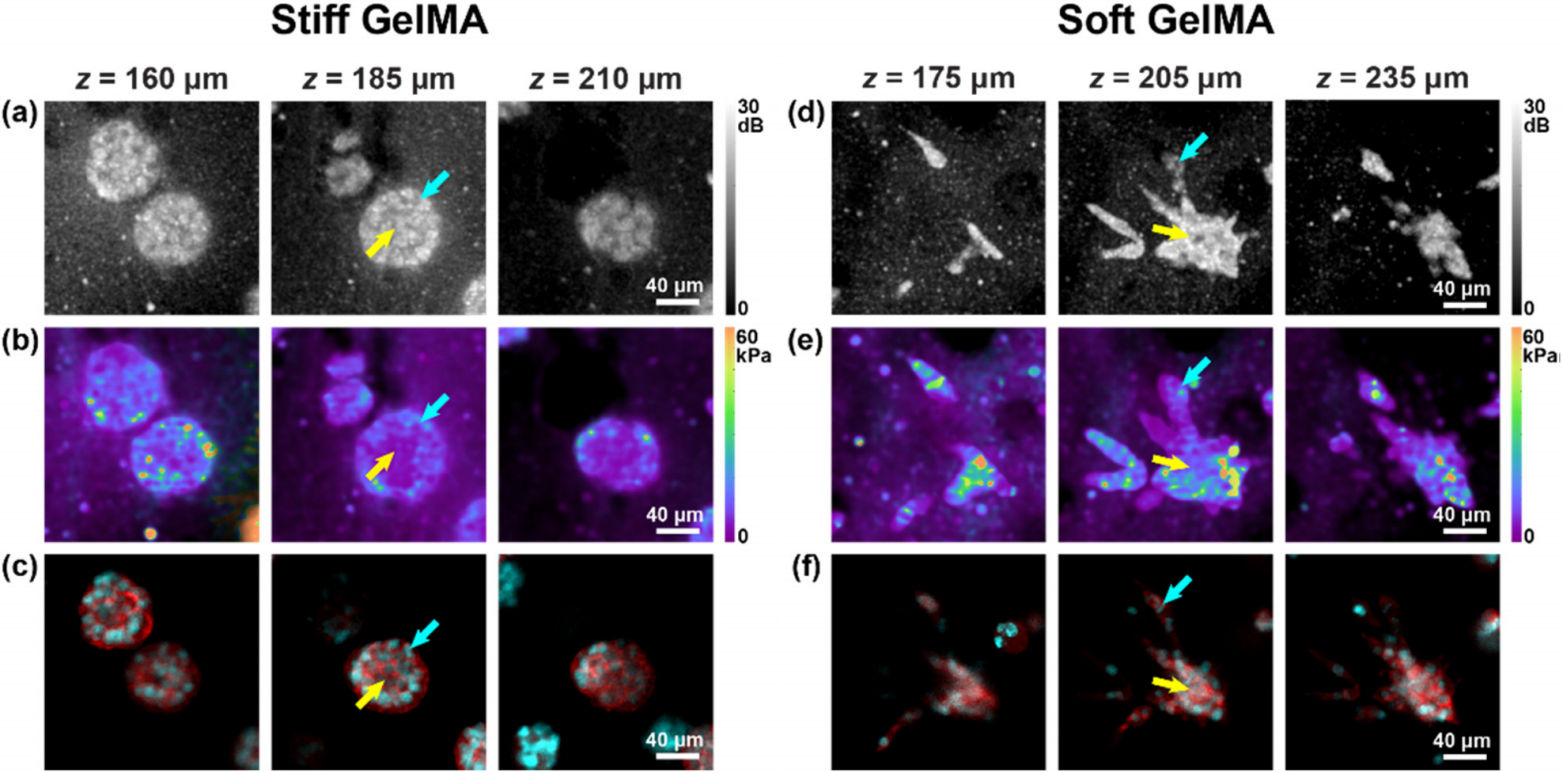
Multimodal mechano-microscopy of metastatic breast cancer cell spheroids in stiff and soft GelMA. *En face* images of [(a) and (d)] OCM SNR, [(b) and (e)] Young's modulus, and the co-registered [(c) and (f)] fluorescence labeled with nuclear (cyan) and membrane (red) fluorescent dyes, presented at three annotated depths, corresponding to the top, middle, and bottom cross-sectional planes of the cell spheroids. Yellow and cyan arrows in (a)–(f) indicate the core and periphery of the cell spheroids, respectively.

In Fig. 4, examples of metastatic spheroids cultured in both the stiff [Figs. 4(a)–4(c)] and soft [Figs. 4(d)–4(f)] hydrogels are presented. Interestingly, metastatic spheroids in a stiff environment do not present cell dissemination. Only when such spheroids are in a softer environment can early metastatic dissemination be observed. Metastatic spheroids in stiff hydrogels present a similar spatial distribution of Young's modulus to that of the non-metastatic spheroids; however, they demonstrate an overall lower Young's modulus [Fig. 4(b)], as indicated by the quantitative comparison shown in Table I. In addition, the peripheral cell membrane and ECM border exhibit disorganization, and an overall higher density of nuclei in the core [Figs. 4(a)–4(c)], compared to their non-metastatic counterparts. Distinct to all the other cases, dissemination of metastatic spheroids in a soft environment is contrasted by a softening of the peripheral cells (7.67 ± 0.71 kPa) compared to the core (11.8 ± 2.73 kPa) and an associated decrease in peripheral nuclear density [Figs. 4(d)–4(f)], possibly induced by increased nuclear volume in disseminating cells.^41^ Although cell invasion has been suggested in stiffer and more constricting ECM environments, the associated mechanisms are still poorly understood in 3D contexts, emphasizing the present need to disentangle the roles of ECM pore size, density, and local Young's modulus from the mechano-phenotype.^1,5^ Interestingly, a similar reduction in invasive phenotypes in stiff 3D hydrogels has been observed in 2D images of Brillouin frequency shift and, also, with fluorescent elastic beads used as compressive stress sensors,^42^ giving promise to 3D models unifying our understanding of cancer invasion.

**FIG. 5.**
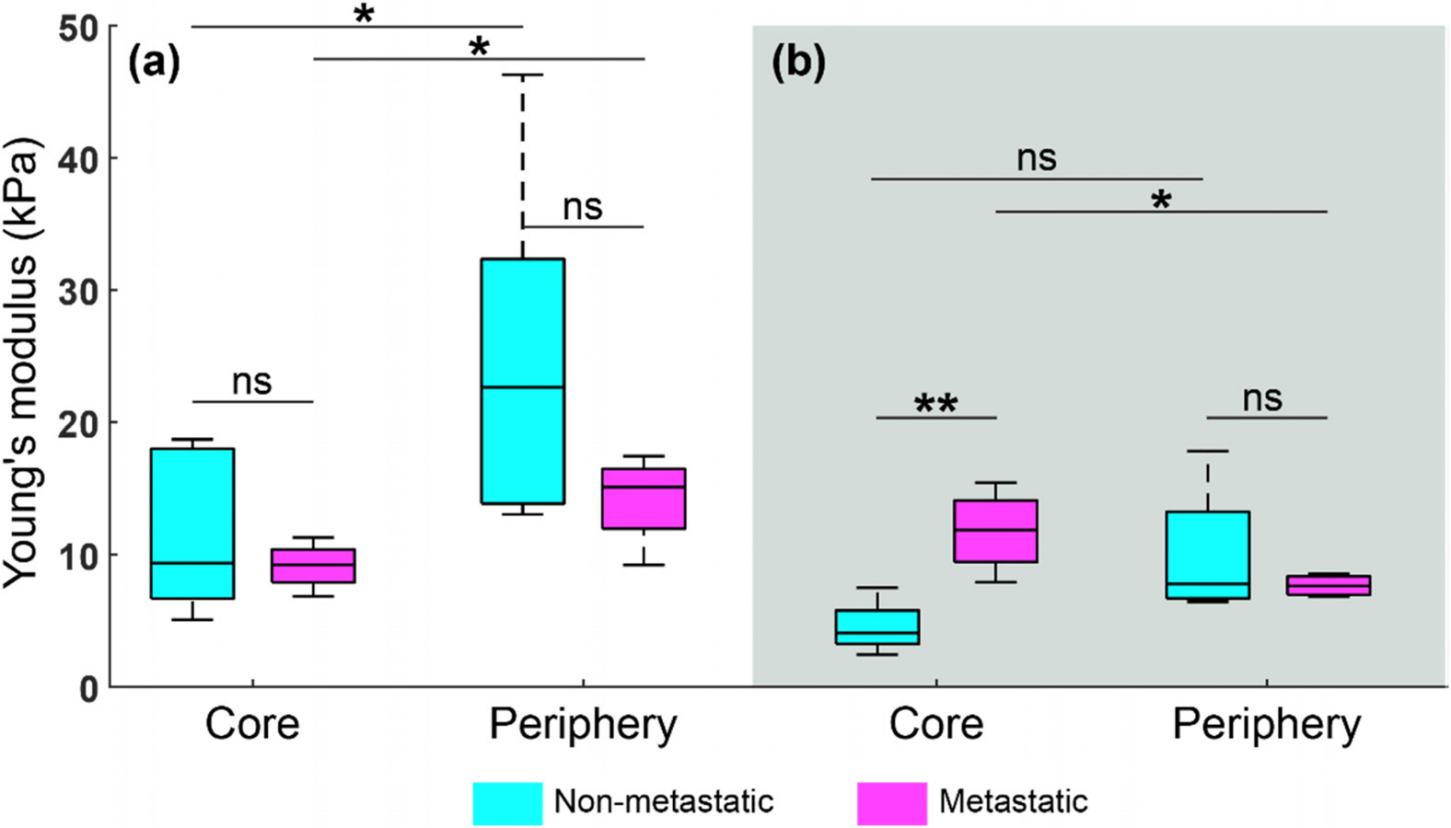
Statistical comparisons between Young's modulus at the core and periphery regions in non-metastatic and metastatic spheroids cultured in (a) stiff and (b) soft hydrogels. The statistical analysis was performed using a two-sample *t*-test with sample size *n* = [6, 4, 6, 4, 4, 4, 4, 4] from left to right in each boxplot, showing no statistical significance (ns) between the cores of non-metastatic and metastatic spheroids in stiff hydrogels, between the peripheries of non-metastatic and metastatic spheroids in both soft and stiff hydrogels, as well as between the core and periphery in non-metastatic spheroids in soft hydrogels, but statistical significance between the core and periphery in both non-metastatic (*p* = 0.038 < 0.05, ^*^) and metastatic spheroids (*p* = 0.043 < 0.05, ^*^) in stiff hydrogels, between the core and periphery in metastatic spheroids (*p* = 0.045 < 0.05, ^*^) in soft hydrogels, as well as between the cores of non-metastatic and metastatic spheroids (*p* = 0.0088 < 0.01, ^**^) in soft hydrogels. In each boxplot, the central mark indicates the median, the top and bottom edges of the box indicate the 25th and 75th percentile, respectively, and the top and bottom whiskers indicate the maximum and minimum values, respectively.

The statistical analysis presented in Fig. 5 suggests significant differences in the Young's modulus between the core and periphery of metastatic spheroids in both soft and stiff hydrogels. Specifically, our results suggest that the core of metastatic spheroids in soft hydrogels is significantly stiffer (*p* = 0.045 < 0.05, ^*^) than the periphery [Fig. 5(b)], which is opposite to the case where the core of metastatic spheroids in stiff hydrogels is significantly softer (*p* = 0.043 < 0.05, ^*^) than in the periphery [Fig. 5(a)]. This statistical trend suggests that the initiation of cell invasion, characterized by the dissemination of metastatic spheroids [Figs. 4(d)–4(f)], is linked with the Young's modulus of the ECM, while the increased matrix stiffness elevates mechanical confinement that impeded the invasion, which is similar to the findings reported in a recent study using Brillouin microscopy demonstrated on both MCF-7 and MDA-MB-231 models.^42^ These preliminary results highlight how the microenvironment stiffness can regulate cancer invasion in a 3D context. For non-metastatic spheroids, the analysis suggests that the core is significantly softer (*p* = 0.038 < 0.05, ^*^) than the periphery in stiff hydrogels [Fig. 5(a)], which is likely due to increased deposition and remodeling of ECM proteins by the cancer cells.^39,40^ This is consistent with the findings reported in a previous study using a low-resolution OCE technique.^20^ We hypothesize that the no statistical significance (ns) between the Young's moduli of the core and periphery of non-metastatic spheroids in soft hydrogels is due to the lack of external forces being exerted on the non-metastatic spheroid. As a result, there are no mechanical cues to encourage cell stiffening/softening, nor are the cells attempting to infiltrate away from the main spheroid body. In addition, our results suggest that the core of metastatic spheroids is significantly stiffer (*p* = 0.0088 < 0.01, ^**^) than that of non-metastatic spheroids in soft hydrogels [Fig. 5(b)]. The statistically different mechanical contrast is because of the *in situ* behavior of metastatic models distinct to non-metastatic models in a soft microenvironment, which could be relevant to cancer progression *in vivo*. In summary, the results of statistical analysis shown in Fig. 5 are consistent with the distinctive patterns highlighted in Figs. 3 and 4.

## III. DISCUSSION

In this paper, we have introduced multimodal mechano-microscopy, a unique imaging platform that provides micro-scale, 3D images of the structure, biomechanics, and biochemistry of living tumor spheroids encapsulated in GelMA hydrogels. To demonstrate this important new imaging capability in the fields of biology and mechanobiology, we demonstrate multimodal mechano-microscopy on established cancer models, i.e*.,* non-metastatic (MCF-7) and metastatic (MDA-MB-231) cancer cell spheroids, cultured in both stiff and soft environments. Importantly, through direct comparison between metastatic breast cancer cell spheroids in stiff and soft GelMA, we have demonstrated that cancer invasion highlighted by the dissemination of metastatic cell spheroids is regulated by the ECM stiffness and is facilitated by softening of the matrix and metastatic cancer cells in the periphery. Given the importance of reconciling these properties in most *in vitro* models, we believe that our imaging platform will find broad application in other areas, including in imaging other types of spheroids, as well as single cells embedded in biomaterials and tissue organoids. Additionally, analogously to related OCE techniques, multimodal mechano-microscopy can be used to provide insight into tissue mechanics, with higher spatial resolution in 3D than is currently possible.

In this study, the GelMA hydrogels used are approximately elastic, with cross-linking chemistry that creates micro-scale pores,^43^ which provides a mechanical confinement for outward cell expansion.^44,45^ In fact, the restricted growth of non-metastatic spheroids indicated by the decreased volume size observed in the experiments is consistent with our previous findings using a lower-resolution OCE system.^20^ This is attributed to the reduced pore size of GelMA hydrogels used, which is inversely related to the stiffness, prohibiting the growth of non-metastatic spheroids, and, moreover, restricts the invasion of metastatic spheroids, which is observed, for the first time, in the context of elasticity imaging in 3D. In a related study by Mahajan *et al.*,^42^ MCF-7 and MDA-MB-231 spheroids were also studied using fluorescence microscopy and Brillouin microscopy. Similarly to our study, these spheroids were cultured in 3D polyethylene glycol (PEG) heparin hydrogels, which were cultured for 14 days rather than 6–8 days. Although the authors did not compare the Brillouin frequency shift (interpreted as a mechanical property that is loosely linearly related to Young's modulus) at the core and periphery of the spheroids, they showed that MCF-7 spheroids embedded in stiff hydrogels demonstrate elevated Brillouin frequency shift, indicative of increased longitudinal modulus. This is similar to our findings that MCF-7 spheroids embedded in stiff GelMA demonstrate higher overall elasticity. Moreover, they showed that MDA-MB-231 spheroids embedded in stiff hydrogels are less aggressive and demonstrate higher Brillouin frequency shift compared to spheroids embedded in softer hydrogels, which show lower Brillouin frequency shift and higher dissemination. These findings are consistent with our results.

In general, a comparative analysis between mechano-microscopy and existing techniques would provide validation of our imaging system. However, this is challenging as there is a paucity of available techniques that can directly map 3D elasticity with sub-cellular resolution. While Brillouin microscopy can map sub-cellular mechanical properties in 3D, the measured longitudinal modulus cannot be directly related to Young's modulus without *a priori* knowledge of Poisson's ratio. Furthermore, the Brillouin frequency shift depends not only on the Young's modulus of the sample but also on the water content within the sample. These factors limit the effectiveness of a comparative analysis using Brillouin microscopy. Nevertheless, in a future study, one could integrate Brillouin microscopy and mechano-microscopy to enable multimodal quantitative optical elastography, which would provide insight into cell mechanobiology on the correlation between Young's modulus and longitudinal modulus in tumor spheroids.

In this study, we have demonstrated that CFM, integrated with mechano-microscopy, enables co-registered OCM, mechano-microscopy, and fluorescence images to relate sub-cellular structures with localized regions of Young's modulus within spheroids in 3D. Notably, our images revealed a strong correlation between higher nuclear density and elevated Young's modulus within the spheroids. This observation is consistent with existing literature that the nucleus is significantly stiffer than the surrounding cytoplasm.^46–48^ Additionally, an important point to consider is that mechano-microscopy requires spatial averaging of deformation over a volume of 5 × 5 × 15 *μ*m^3^ to minimize the effect of dark speckle on the accuracy and precision of measurements,^49^ which, although providing sub-cellular resolution, is lower resolution than that of confocal fluorescence microscopy (0.5 × 0.5 × 8 *μ*m^3^). This difference in spatial resolution, in addition to other effects, such as the sequential nature of the imaging method employed, makes exact co-registration of the two modalities challenging. In future studies, we plan to implement a hardware-based approach to eliminating speckle from OCM, by averaging a number of independent speckle realizations created by an external mechanism, such as a mechanical load^50^ or a diffuser.^51^ In addition, to minimize the time delay between the different imaging modalities, we will implement a scanning protocol to provide simultaneous acquisition of mechano-microscopy and CFM. An additional limitation for time-lapsed imaging in the current setup is the lack of control of environmental factors, such as temperature, humidity, CO_2_, and O_2_. To address this, the imaging platform could be developed toward high-throughput mechanical phenotyping and drug screening by integrating a customized stage-top incubator,^52^ combined with an automated wide-field scanning system^53^ to accelerate the speed of volumetric imaging over the entire sample area.

Staining cell nuclei and membranes in live spheroids effectively visualizes intra-spheroidal morphological variation and sub-cellular functional deformation associated with mechanical phenotypes of cancer spheroids. We believe that the variation of Young's modulus depends on the stiffness of sub-cellular organelles, as well as the arrangement of live cells interacting with each other and ECM during cancer development. Although nuclei density could be a possible contributing factor, there are other contributors to the Young's modulus variation in the spheroid such as cancer microenvironment and growth biology governing the cell-to-cell and cell-to-ECM contacts. In future studies, further insight could be gained by staining other important organelles associated with mechanotransduction, such as actin filaments and focal adhesions, that drive the mechanobiology of cancer cells.^54,55^ In addition, we consistently observed greater nuclear density in the spheroid periphery than in the core in the fluorescence images (e.g*.,* MCF-7 spheroids in Fig. 3), corresponding to the elevated Young's modulus in the periphery. The pattern of nuclear distribution is possibly due to the initiation of hypoxic conditions and necrotic-like phenotypes in the core at the stage of the spheroid growth. However, further investigation is required to validate this hypothesis, for example, using HIF-1α,^56^ in a future study. For long-term monitoring of spheroids in customized stage-top incubators, suggested above, one can transfect cells with fluorescent proteins. This would provide stable and long-lasting fluorescent markers integrated within the cells' natural protein structures.^57^ Furthermore, incorporating extra excitation wavelengths, such as 561 nm, in addition to 405 and 488 nm channels, would provide greater flexibility for using a wider range of fluorescent probes.

The central wavelength of the light source used in our system is ∼800 nm, providing a measured OCM axial resolution of 1.4 *μ*m in air. It has been reported that visible-light OCM, where the central wavelength is in the visible portion of the electromagnetic spectrum, can provide sub-micrometer axial resolution.^58^ However, a disadvantage of this approach is the high optical attenuation at these lower wavelengths, which limits the penetration depth in turbid samples. For this reason, we choose a longer wavelength, trading off axial resolution for increased penetration depth, enabling us to image through entire tumor spheroids. Separately, the OCM system demonstrated in this study utilizes a high numerical aperture (NA) microscope objective to achieve sub-micrometer lateral resolution (detailed in Sec. IV A) that is required to reveal intra-spheroidal structures. As a trade-off, the high NA beam significantly decreases the depth of field, which is ∼10–15 *μ*m around the focal plane. To address this, in this study, we implemented dynamic focusing^59^ to extend the depth of field for capturing the entire spheroid (in thickness of 50–60 *μ*m) by acquiring multiple volume scans at different axial locations, which is described in Sec. IV B. To broaden the applications of mechano-microscopy to other fields, such as in tissue engineering where the mechanical characterization of larger-scale biological samples (e.g., organoids and biomaterials) with sub-cellular spatial resolution could provide further insights, the imaging capability of mechano-microscopy could be further enhanced by improving aspects of OCM beam illumination and detection. For example, it has been demonstrated that the trade-off between imaging depth and lateral resolution can be improved using Bessel beam illumination.^60^ While use of Bessel beam typically reduces OCT/OCM sensitivity, compared to Gaussian beams used in this study, an extended depth of field of 100 *μ*m and sub-2 *μ*m lateral resolution can be achieved with a single acquisition and has been demonstrated by our group.^61–63^ In addition, the use of a metalens has been demonstrated in OCM to ease the trade-off with high imaging speed by flexibly manipulating the phase, amplitude, and other properties of the wavefront.^64^ Developing metasurface-based mechano-microscopy may mitigate temporal effects resulting from cell movement on elasticity accuracy by accelerating the acquisition speed.

To estimate Young's modulus of the sample, the mechanical model used in mechano-microscopy, as is the case for most elastography techniques, requires simplifying assumptions on the nature of the sample's properties and its deformation. In this study, to minimize the hyperelastic effect of the sample on the measurements of Young's modulus,^65^ a minimum pre-strain (<5%), i.e., a bulk strain applied prior to the micro-scale actuation, was used to ensure uniform contact between the sample, layer, and compression plates. Furthermore, to reduce viscoelastic effects, the imaging protocol was carefully tailored by encapsulating cell spheroids into GelMA that exhibits low viscoelasticity,^66^ and the samples were deformed at a quasi-static loading frequency^17^ to ensure the instantaneous elastic strain was measured. However, instead of minimizing these effects on the accuracy of elasticity measurements, in future work, we believe that, given the important role of viscoelasticity, viscosity, and pore size of the matrix, in addition to elasticity, on the migration of cancer cells,^67^ it is important to develop a multi-parametric mechano-microscopy system for more complete characterization of tumor spheroids in a 3D microenvironment. For example, a related lower-resolution OCE technique developed by our group has demonstrated viscoelasticity imaging of freshly excised rat gastrocnemius muscle.^68^ This technique could be adapted for mechano-microscopy. In addition, while it is difficult to resolve the pore size in GelMA hydrogels using OCM, information of porosity could be determined from the Poisson effect by measuring both the axial and non-axial components of displacement and the resulting strain using 3D displacement estimation^69,70^ and strain tensor imaging,^71,72^ which has been demonstrated in previous studies of ultrasound elastography.^73^ Furthermore, in any compression elastography technique, including mechano-microscopy, the accuracy of elasticity estimation is inherently limited by local stress variation along the boundary of stiff features due to mechanical coupling and geometry. Our previous work demonstrated the effect of stress nonuniformity on phantoms containing stiff inclusions of varying elasticity embedded in a softer background material. The results of that work suggested that this effect introduced relatively minor errors for mechanical contrast less than a factor of 10.^74^ In the case of cell spheroids, the mechanical contrast ratio between spheroid and GelMA is ∼3–6, which is relatively small. In addition, for cell spheroids with round and smooth edges, the stress-related effect should be reduced compared to the cubic inclusions with sharp corners and flat boundaries. Hence, we believe that the measured elasticity within spheroids is accurate in mechano-microscopy. Nevertheless, a systematic investigation enabled by finite element simulations could be performed in the future to analyze the effect of elasticity contrast and feature geometry on the accuracy of elasticity estimated using mechano-microscopy. Alternatively, to retrieve a sample's intrinsic mechanical properties that are largely independent of the sample geometry, stress distribution, and boundary conditions, computational approaches that make less sample-related assumptions may provide more accurate solutions to the inverse elasticity problem in elastography. Dong *et al.* demonstrated an iterative method that incorporates adjoint-based algorithms to reconstruct the spatial distribution of elasticity without assumptions regarding uniformity of stress or mechanical properties of the sample in the context of compression elastography.^75,76^ However, these approaches are typically computationally demanding and do not provide an estimate of the uncertainty in the prediction of elasticity due to the deterministic nature of the algorithm.

Visualizing stiffness at the cellular and sub-cellular level has the potential to provide insight into the genesis and progression of disease. We believe that mechano-microscopy could be a valuable addition to a family of existing techniques to map stiffness across length scales from whole organs to sub-cellular structures. For example, medical imaging techniques, such as ultrasound elastography^77^ and magnetic resonance elastography,^78^ have been widely adopted to map the macro-scale variation in the stiffness of entire tissues in clinical settings. OCE has shown promise in the subsurface imaging of tissues and biomaterials on the micro-scale between that of cells and tissues.^14^ On the cellular scale, AFM provides 2D elasticity imaging of sub-cellular structures on the sample surface.^6^ Importantly, we believe, beyond applications in cell mechanobiology, mechano-microscopy, with spatial resolution between that of OCE and AFM, could provide important insight on the link between tissue stiffness and disease with sub-cellular spatial resolution and depth sectioning up to hundreds of millimeters. In fact, ultrahigh-resolution OCE,^61^ an early version of mechano-microscopy, but with relatively low spatial resolution (∼15 *μ*m isotropic spatial resolution), has been demonstrated to image cellular scale stiffness of mouse aorta through the entire depth of an intact aortic wall.^62^ Another advantage of mechano-microscopy is that, as the loading direction is aligned with the optical axis, it is well-suited to implementation in compact ultrahigh-resolution imaging probes, such as micro-endoscopes,^63^ to overcome the limit of depth penetration, which is challenging to implement in other sub-cellular imaging techniques (e.g., AFM and Brillouin microscopy). The technical specifications of mechano-microscopy present new opportunities for a broader translation to biomedical applications than is possible using existing techniques.

In summary, most solid tumors, including breast tumors, exhibit dysregulated extracellular matrix deposition leading to tissue stiffening, which is associated with poor prognosis. This makes the mechanical changes in tumor microenvironment as an emerging hallmark of cancer. Mechanotransduction plays a pivotal role in how cells interact with their surroundings, including the extracellular matrix and neighboring cancer cells. The primary interfaces for these interactions are integrin-mediated focal adhesions for cell–matrix interactions and cadherin-mediated adherens junction for cell–cell interactions. To understand how cancer cells sense their surrounding tumor microenvironment at a sub-cellular level (such as single focal adhesions or adherens junctions), it is essential to investigate the sub-cellular mechanical properties within both the spheroids and the surrounding hydrogel. This approach will facilitate direct comparison of sub-cellular information obtained from immunocytochemistry of proteins of interest with local mechanical stimuli. Multimodal mechano-microscopy represents a significant advancement in the understanding of cancer mechanobiology, by facilitating 3D and sub-cellular characterization of cancer spheroid models encapsulated within stiffness-tunable hydrogels. Beyond this example, mechano-microscopy is ready to explore a host of cancer types and TME models in 3D and is well poised to propel mechanobiology toward the quantification and specificity needed to understand complex biophysical processes in disease and development.

## IV. METHODS

### A. Multimodal mechano-microscopy

**FIG. 6.**
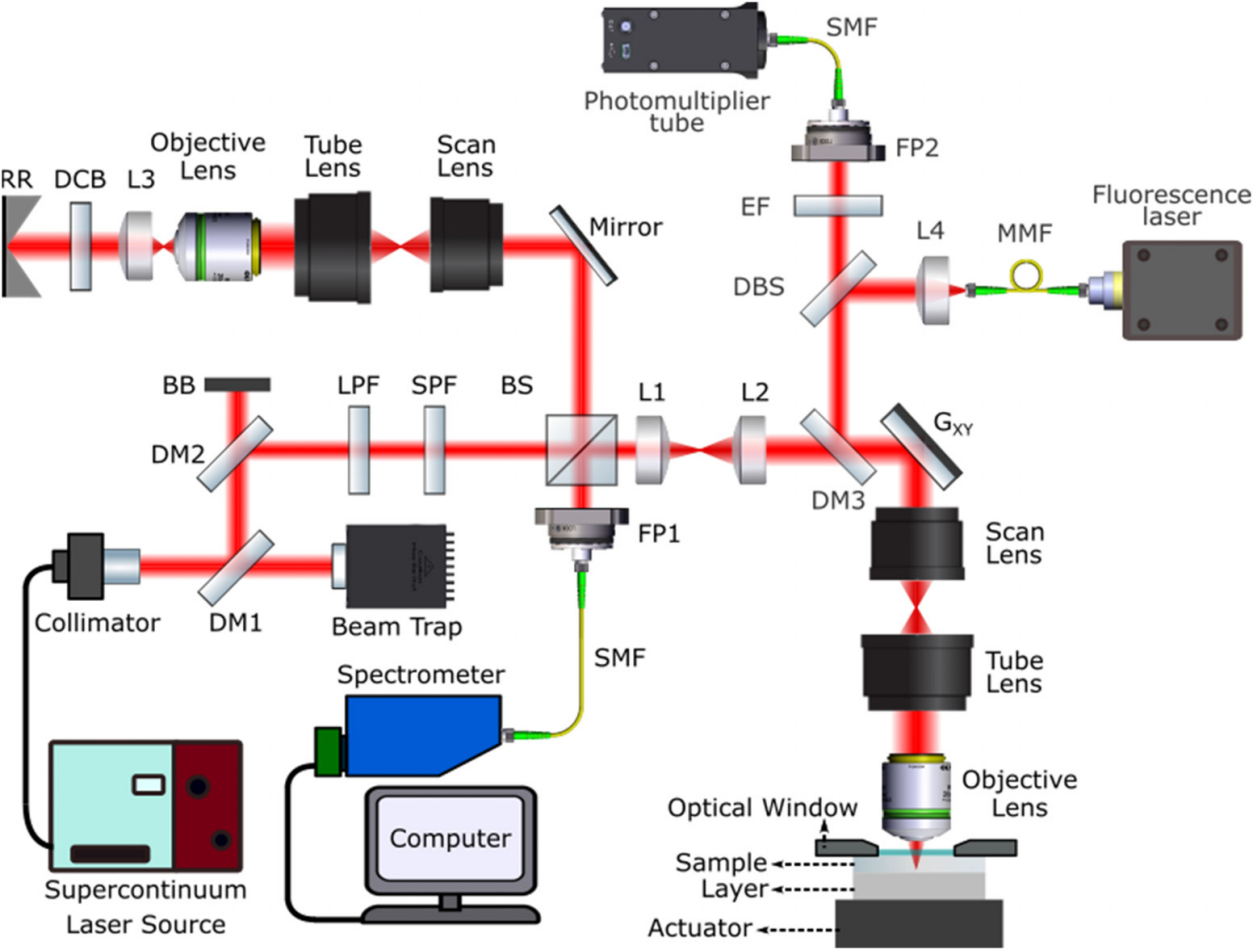
Schematic diagram of the multimodal mechano-microscopy system. RR: reference reflector, DCB: dispersion compensation block, L: lens, BS: beam splitter, SPF: short-pass filter, LPF: long-pass filter, BB: beam block, DM: dichroic mirror, FP: fiber port, SMF: single-mode optical fiber, G_xy_: *xy* galvanometer mirrors, DBS: dichroic beam splitter, MMF: multi-mode optical fiber, EF: edge filter.

Multimodal mechano-microscopy integrates high-resolution interferometric detection of OCM, a high-resolution variant of OCT,^27^ with compression OCE^31^ and CFM. A schematic diagram of the imaging system is shown in Fig. 6. The system uses a supercontinuum laser (SuperK Extreme EXW-4 OCT, NKT Photonics, Denmark), whose output was shaped to a spectral range of 650–950 nm using a combination of a long-pass dichroic mirror (DMLP950, Thorlabs Inc., USA), a short-pass dichroic mirror (DMSP650, Thorlabs Inc., USA), a long-pass filter (FELH0650, Thorlabs Inc., USA), and a short-pass filter (FESH0950, Thorlabs Inc., USA). This spectrum corresponded to a full-width at half-maximum (FWHM) bandwidth of ∼250 nm, providing a measured OCM axial resolution of 1.4 *μ*m in air. The system was implemented as a Michelson interferometer in a dual-arm configuration with the same optics in the reference beam path to match optical dispersion. A dispersion compensating block was used to account for residual dispersion. The sample arm beam was expanded to fill the entrance pupil of the objective lens (20 × 0.75 NA, CFI Plan Apo, Nikon) providing a measured lateral resolution of 0.5 *μ*m. Scanning was achieved using a 2D galvanometer system (GVSM002-EC/M, Thorlabs Inc., USA).

A sample with a thickness of ∼400–500 *μ*m and a 1 mm thick compliant silicone layer were compressed between the coverslip used as an optical window and an annular piezoelectric actuator (Piezomechanik GmbH, Germany). We used a spectrometer comprising a 2048-pixel line camera with a maximum line rate of 130 kHz (Wasatch Photonics, USA) to detect the spectral interference at each *xy* location. The B-scan and volume acquisition times were 20.4 ms and 40.8 s, respectively. The annular actuator was driven in a quasi-static regime with a 24.5 Hz square wave, synchronized with the acquisition of B-scans. Two B-scans were acquired at each *y*-location such that one B-scan was acquired at the unloaded and one at the loaded state. Local displacement was calculated from the phase difference between the loaded and unloaded B-scans and strain was estimated as the gradient of the axial displacement with depth using weighted least squares linear regression.^31^ Strain in the layer was related to stress through knowledge of the stress–strain response of the compliant layer in contact with the sample. By assuming uniaxial stress, the local stress at the surface of the sample was divided by the local strain in the sample to estimate sample elasticity as the tangent modulus, which is equivalent to Young's modulus under the assumption of linear elasticity. An elasticity system resolution of 5 × 5 × 15 *μ*m^3^ (*xyz*) is calculated as the convolution of the OCM system resolution and the FWHM of the signal processing used, i.e., Gaussian smoothing and weighted least squares linear regression.^79^ To minimize the effect of friction on the precision^80^ and accuracy^81^ of the estimated Young's modulus, customized hyaluronic acid (HA) solution was made to improve the lubrication of the contact surface between the window and GelMA hydrogels.^82^

The integrated CFM system comprised two laser lines at 405 and 488 nm as the excitation light source (CNI Lasers, Changchun New Industries Optoelectronics Technology Co., China) and a photomultiplier tube (PMT2101/M, Thorlabs Inc., USA) as the detector. The CFM system used the same galvanometer scanning arm and optics as the OCM system to scan the focal point in the sample. The emitted fluorescence was focused into a single-mode optical fiber, which also acted as the confocal pinhole. The focal plane of the CFM system was aligned with the OCM system to co-register the focal planes of both imaging systems in the sample. The CFM point-spread function (PSF) was measured using small beads, and the resolution was determined from the FWHM of the PSF as 0.5 *μ*m in the lateral direction and ∼8 *μ*m in the axial direction. To obtain a *z*-stack and generate the 3D CFM image presented in Fig. 1(g), the sample mount setup was scanned in *z* in steps of 5 *μ*m. The recorded fluorescence intensities at each scan point were saved into 64-bit, 2D binary images. Fiji software^83^ was used to combine the two channels and multiple focal planes into 3D composite image stacks.

### B. Dynamic focusing to extend the depth of field

Mechano-microscopy uses a high NA microscope objective to achieve sub-micrometer lateral resolution. However, the high NA beam significantly decreases the depth of field, therefore, the imaging depth is limited to ∼10–15 *μ*m around the focal plane. To overcome the limited depth of field and to extend the imaging depth, dynamic focusing was performed by scanning the sample at consecutive axial locations^59^ and acquiring multiple volumetric scans. To generate the 3D visualizations in Figs. 1(f) and 1(g), 11 volumetric scans were acquired at partially overlapping focal depths along the *z*-axis inside the sample within ∼9 min. To vary the focus position in the sample, the sample mount assembly was translated in consecutive steps of 10 *μ*m in air using a motorized translation stage. To generate the Young's modulus image with extended depth of field as shown in Fig. 1(g), Young's modulus was computed for each volumetric scan position, then, an axial Gaussian filter was applied with a peak corresponding to each volume's focal plane. We summed the filtered Young's modulus subvolumes to generate the extended depth of field 3D image. A similar approach was used to generate the 3D-OCM visualization in Fig. 1(f).

### C. Compression optical coherence elastography

To highlight the improved imaging capability of multimodal mechano-microscopy, as shown in Fig. 2, we compared its performance on cell spheroids with compression OCE based on a lower-resolution OCT system demonstrated in a previous study on individual cells in 3D.^17^ We pre-aligned the two systems and translated the sample setup from the multimodal mechano-microscopy to OCE, and we performed consecutive scans of the same sample under the same loading conditions. The OCT system used is a fiber-based spectral-domain OCT system (Telesto 220, Thorlabs Inc., USA) that has a spectral bandwidth of 170 nm and a central wavelength of 1300 nm. The measured axial and lateral resolutions (FWHM) are 4.8 and 4.4 *μ*m, respectively. Similar to mechano-microscopy, signal processing in OCE includes the combination of Gaussian smoothing and weighted least squares linear regression, resulting in an isotropic elasticity system resolution of 35 *μ*m, calculated as the convolution of the OCT system resolution and the FWHM of the signal processing used.

### D. Cell culture, hydrogel fabrication, and cell encapsulation

MCF-7 (non-metastatic breast cancer) and MDA-MB-231 (metastatic breast cancer) cells were cultured using T-75 flasks (Conoco) and maintained at 37 °C with 5% CO_2_ until 80% confluency was reached. Routine cell culture involved replacement of media every 2 days. This consisted of high glucose Dulbecco's Modified Eagle Medium (hg-DMEM:Gibco), with 1% (v/v) antibiotic-antimycotic (anti-anti; Gibco) and 10% (v/v) fetal bovine serum (FBS; Gibco). To passage MCF-7 and MDA-MB-231 cells, 2 ml of Ethylenediaminetetraacetic acid solution (Sigma) (w/v) was added and the resultant cell suspension was retrieved and added to 5 ml of Hg-DMEM in a 15 ml tube and centrifuged at 1200 rpm for 5 min. The supernatant was then discarded, the cell pellet resuspended in 1 ml of Hg-DMEM. Cells were then populated using a hemocytometer and trypan blue (Sigma). Cells were seeded at a density of 2000 cells/gel within pre-warmed precursor GelMA solution. The cell suspension was pipetted through a P200 at least 10 times to ensure cell aggregates were dismantled. We note that we aimed to embed cells as single cells so that spheroid formation was a result of proliferation, thus generating spheroids of relatively consistent size.

GelMA was synthesized by methacrylation of gelatin as described previously.^84^ Briefly, 10 g gelatin (G1890, Sigma-Aldrich) was dissolved in 100 ml of de-ionized water at 60 °C under constant stirring in a round-bottom flask. Then, 8 ml of methacrylic anhydride (276685, Sigma-Aldrich) was added dropwise to the gelatin solution stirred for 2 h at 60 °C. The gelatin-methacrylic anhydride solution was then mixed with preheated de-ionized water at 60 °C and maintained for 1 h. The resulting solution was dialyzed in de-ionized water at 55 °C for 7 days using a dialysis membrane (MWCO, approximately 12–14 kDa, Spectrum Laboratories). Subsequently, the solution was filtered through 6 *μ*m-sized pores qualitative filter paper (TY1-110, ADVANTEC), lyophilized, and stored at −20 °C.

Lyophilized GelMA was initially placed in a desiccator vacuum with an attached filter cap (SteriFlip 0.22 *μ*m, Millipore) to remove residual moisture. Following this, GelMA was weighed and dissolved in sterile Dulbecco's Phosphate Buffered Saline (DPBS, Gibco) to make a 9% (w/v) GelMA solution. Additionally, a powdered photoinitiator, Irgacure-2959 [2-Hydroxy-4'-(2-hydroxyethoxy)-2-methylpropiophenone, Sigma-Aldrich] dissolved in absolute ethanol was added to the solution at a final concentration of 0.1% (w/v). The solution was then protected from light and stored at 4 °C overnight prior to use. On the day of encapsulation, the GelMA precursor solution was warmed to 37 °C in a water bath for 1 h. MCF-7 and MDA-MB-231 cells were resuspended in pre-warmed GelMA at 2000 cells/hydrogel. The cell-laden GelMA was then pipetted into circular 5 mm diameter, 300 *μ*m thick polydimethylsiloxane (Wacker Chemie AG, Germany) molds atop a dichlorodimethylsilane-treated (Sigma-Aldrich) glass slide and covered with a methacrylated [3-(trimethoxysilyl) propylmethacrylate, Sigma-Aldrich] circular coverslip (10 mm diameter, Trajan). The cell-laden GelMA was then exposed to UV light (365 nm at ∼2.5 mw/cm^2^, Vilber ECX-F20.L) for 15 or 60 s, respectively, to produce soft or stiff gels. MCF-7 and MDA-MB-231 spheroids were cultured for 16 (Fig. 3) and 6–8 (Fig. 4) days, respectively, to allow for adequate spheroid growth.

### E. Atomic force microscopy

An MFP-3D atomic force microscope (Asylum Research) equipped with 200 *μ*m long gold-coated pyramidal-tipped pyrex-nitride cantilevers (PNP-TR, NanoWorld) was used to measure GelMA surface stiffness. Each sample was indented at five sites that were consistent between samples. Indentations were performed in triplicate at 2 nN while samples were immersed in PBS. Custom code in Igor Pro was used to analyze the linear portion of contact-generated force curves to derive Young's modulus.

### F. Cell staining protocol

The GelMA containing the cell spheroids, attached to coverslips, were kept on a 12-well plate in normal growth medium at 37 °C 5% CO_2_ and stained just before imaging. CellMask Green (C37608, ThermoFisher) was mixed with 0.5 ml of growth medium in 1:200 dilution and Hoechst 33342 was added in 10 *μ*M final concentration. The growth medium in the sample well was replaced with the dye mix, and the sample was incubated at 37 °C for 20 min. The sample was then washed twice with warm PBS and returned to normal growth medium. The samples were imaged within 2 h of staining.

### G. Segmentation method

Young's modulus in the core and periphery regions was determined by masking these regions in structural OCM volumes and then applying the masks to the corresponding Young's modulus. The structural volumes were normalized to a range of 0–1 and then binarized with a threshold of 0.5. Regions that were disconnected from the main spheroid were removed and holes were filled to produce a single contiguous mask of each spheroid. Edge-smoothing was performed by convolving a 20 × 20-pixel (40 × 40 *μ*m^2^) kernel of ones with the initial binary mask, then thresholding out all values below 0.95 to create a smoothed binary mask. The centroid of each mask was determined by calculating the center of mass where each pixel in the binary mask was uniformly weighted. Each pixel was categorized according to its proximity to the centroid. After automatic segmentation of the whole spheroid, we further manually segmented the core and periphery guided by the corresponding OCM images. We define the core as the area pertaining to the central area of the spheroid corresponding to ∼50% of the spheroid diameter and the periphery as the outer area corresponding to ∼50% of the spheroid diameter. Importantly, by using manual segmentation, we were able to better segment the protrusions of metastatic spheroids in soft hydrogels. For these spheroids, segmentation was also performed on an approximately even split based on spheroid diameter, however, we prioritized segmenting all protrusions into the peripheries. The mean Young's modulus corresponding to the core and periphery was calculated in the *en face* planes at the spheroid center. Finally, the Young's moduli (mean ± standard deviation) of the core and periphery were determined for each individual spheroid, which are summarized in Table I.

## Data Availability

The data that support the findings of this study are available from the corresponding authors upon reasonable request.
